# Supplementary material for: TLR2 on blood monocytes senses dengue virus infection and its expression correlates with disease pathogenesis
Source: Nat Commun. 2020 Jun 23;11:3177. doi: 10.1038/s41467-020-16849-7 (PMC7311456; doi:10.1038/s41467-020-16849-7)
Supplement: Supplementary file 1 — Supplementary Information [file 41467_2020_16849_MOESM1_ESM.pdf]

## Supplementary information

# TLR2 on blood monocytes senses dengue virus infection and its expression correlates with disease pathogenesis

Aguilar-Briseño et al.

a

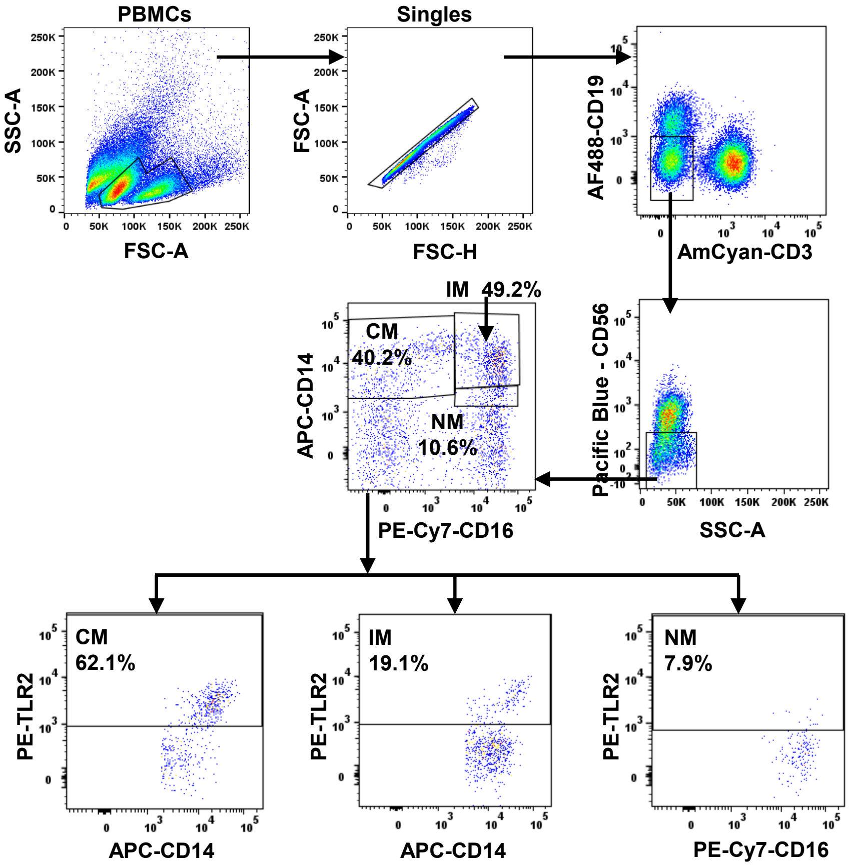


b

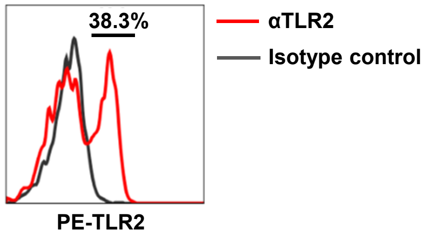


**Supplementary Figure 1. Characterization of monocyte subsets and TLR2 surface expression in monocytes isolated from DENV positive patients. (a)** Gating strategy for the characterization of monocyte subsets and TLR2 surface expression in monocytes from DENV patients as presented in Fig. 1a, 1b, 1c, 1e, Supplementary Fig. 3, 4a, 4b, 10a and 10b. CM= classical monocytes, IM= intermediate monocytes, NM: non-classical monocytes. **(b)** To measure the percentage of cells expressing TLR2, cells were stained with an anti-TLR2 antibody conjugated with PE. An isotype and concentration matched antibody labelled with PE was used as negative control for measuring TLR2 expression. The figure above represents a histogram comparing the anti-TLR2 antibody (red line) and the corresponding isotype control (grey line).

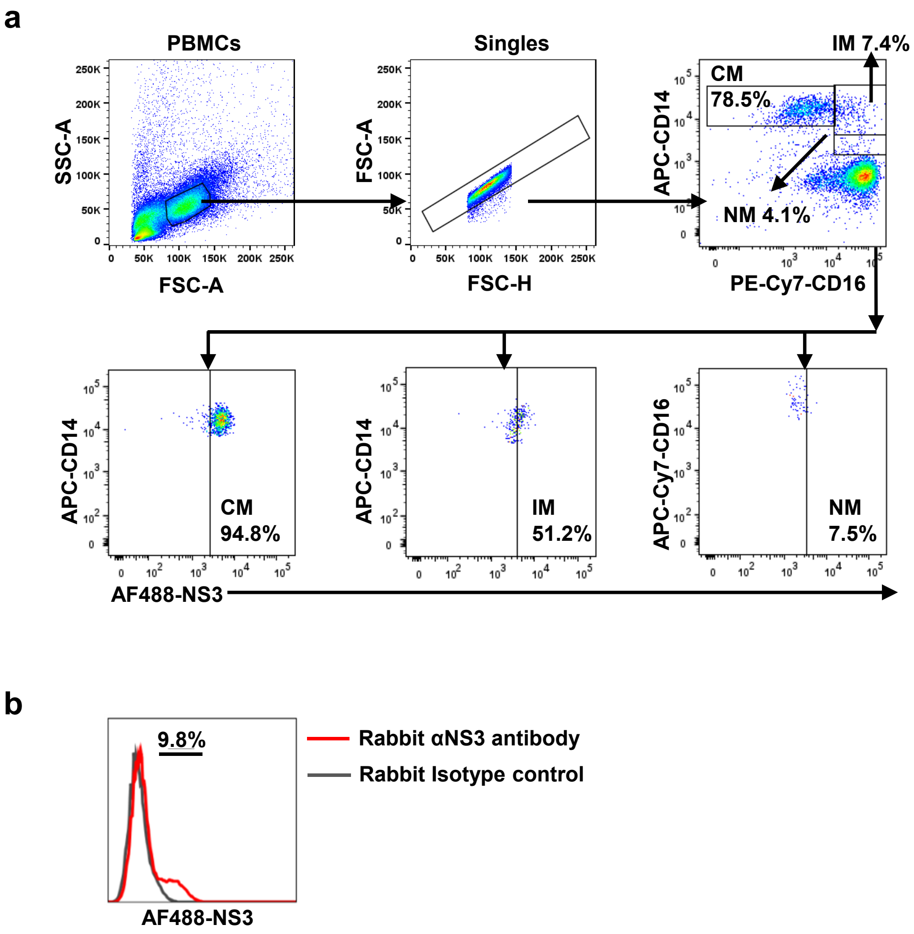


**Supplementary Figure 2. Characterization of DENV infection in monocytes from DENV patients**. (**a**) Gating strategy used for the characterization of DENV infection in patients peripheral blood monocytes as presented in Fig 1d, 2g, Supplementary Fig. 13. (**b**) To confirm the presence of DENV, cells were stained with a rabbit anti-NS3 antibody followed by goat anti-rabbit IgG conjugated with FITC. A non-specific polyclonal rabbit antibody was used as a negative control for staining of DENV infection. The figure above represents a histogram comparing the anti-NS3 antibody (red line) and the isotype control (grey line).

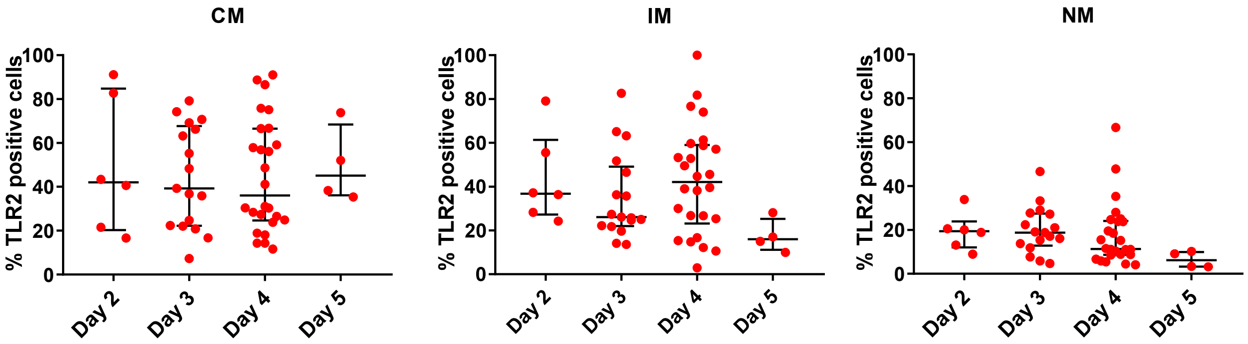


**Supplementary Figure 3. TLR2 expression on monocyte subsets of DENV positive patients**. DENV positive patients were classified according to day post-fever on the day of sample classification (n=53). % of TLR2 positive was determined by flow cytometry. Bars and lines indicate median and IQR. Source data are provided as a Source Data file.

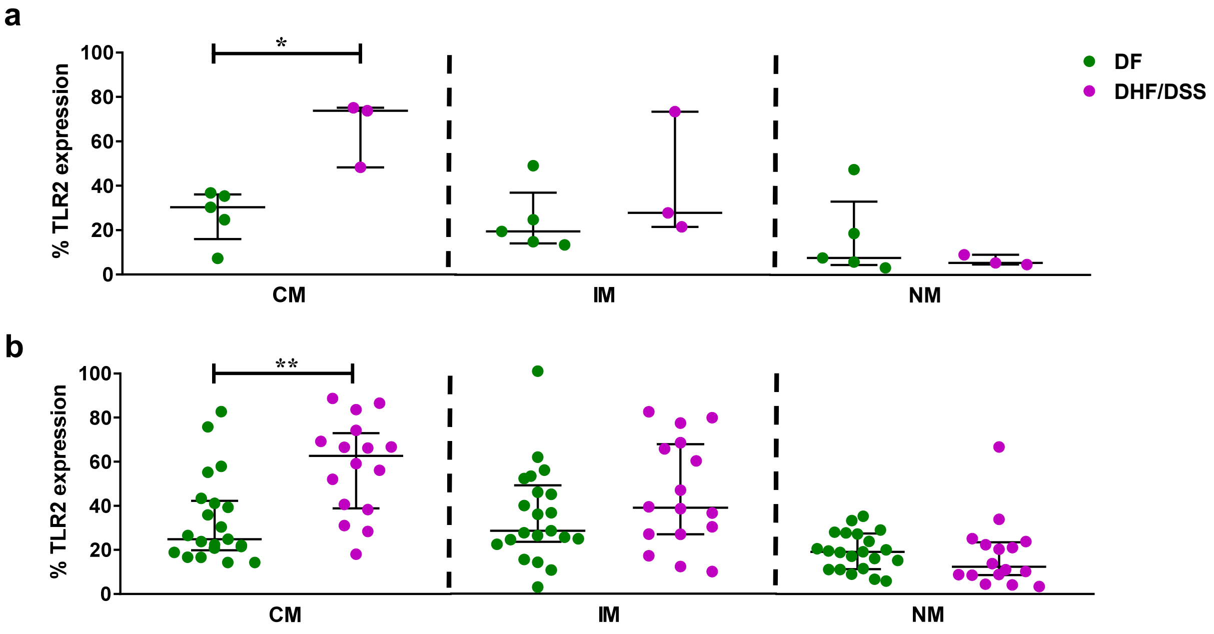


**Supplementary Figure 4. Increased expression of TLR2 in CM from DENV2 and DENV1 positive patients correlates with DENV disease severity. (a** and **b)** PBMCs were isolated from 45 patients undergoing acute DENV serotype 1 infection who developed relatively mild (DF, n=26) or severe (DHF/DSS, n=19) disease. Patients were classified based of infecting serotype, (**a**) DENV2 (two-tailed Mann-Whitney test, * P< 0.05) and (**b**) DENV1 (two-tailed Mann-Whitney test, ** P< 0.01) . Percentages of cells expressing TLR2 were determined for each monocyte subsets stratified by disease severity. Bars represent median with IQR. Source data are provided as a Source Data file.

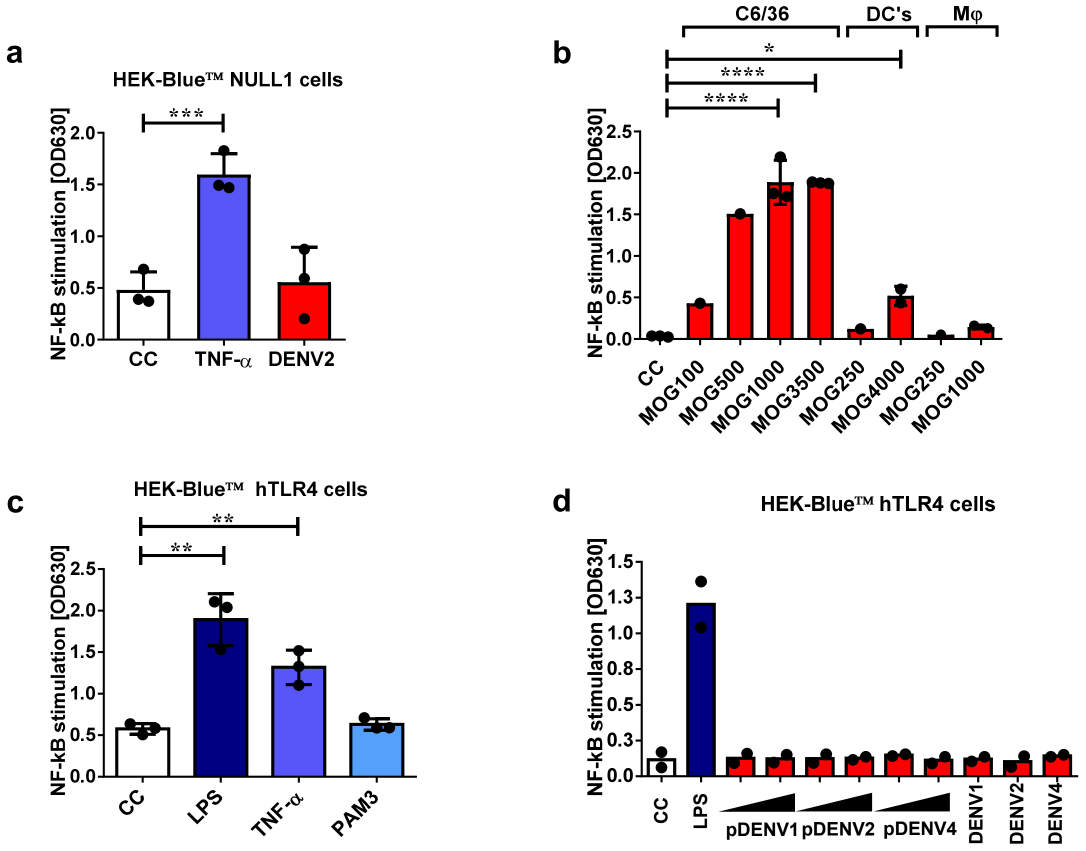


**Supplementary Figure 5. Engagement of DENV is TLR2 specific.** (**a**) HEK-Blue™ Null1 cells were (mock) - treated for 24h with TNF-α (50 ng/mL) and DENV2 (strain 16681, MOI 10), n=3 (paired one-tailed t test, *** P<0.001). (**b**) HEK-Blue™ hTLR2 cells were infected with DENV2 (MOG100, MOG500, MOG1000, MOG3500), DENV2 produced in dendritic cells (DC's, MOG250; MOG4000) and macrophages (Mφ, MOG250; MOG1000) for 24h n=3 (paired one-tailed t test, *P<0.05; ****P; <0.0001). (**c**) HEK-Blue™ hTLR4 cells were treated for 24h with LPS (10 ng/mL) TNF-α (50 ng/mL), PAM3CSK4 (50 ng/mL), n=3 (paired one-tailed t test, **P<0.01) and/or (**d**) MOG3500 and MOG10000 of the purified preparations pDENV1 (strain 16007), pDENV2 (strain 16681), pDENV4 (strain 1036) and with MOG3500 of their respective non-purified preparation, n=2, for 24h. NF-kB stimulation was assessed by QUANTI-Blue™, OD values show the induction of NF-κB. Data represent the mean ± SD. N refers to the number of independent biological experiments. Source data are provided as a Source Data file.

HEK-Blue™ hTLR2 cells

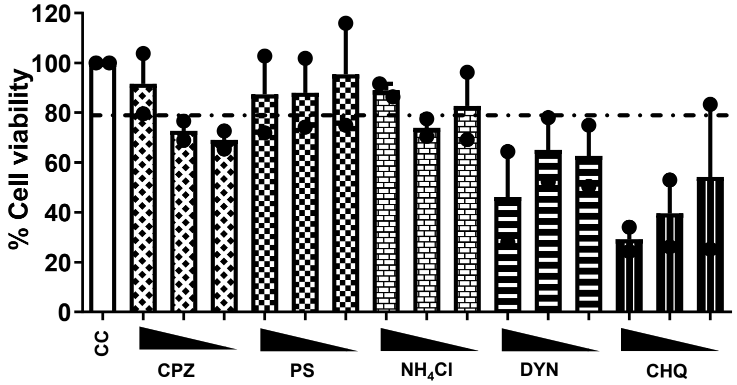


**Supplementary Figure 6. Viability of the HEK-Blue™ hTLR2 cells after treatment with endocytosis inhibitors.** HEK-Blue™ hTLR2 cells were incubated for 24h with chlorpromazine ((CPZ), 20 µg/mL, 10 µg/mL, 5 µg/mL), pitstop ((PS), 30 µM, 20 µM, 10 µM), ammonium chloride ((NH4Cl), 30 µM, 10 µM, 1 µM), dynasore ((DYN), 80 µM, 40 µM, 20 µM) and chloroquine ((CHQ), 50 µg/mL, 20 µg/mL, 5 µg/mL) for 24h (n=2). Cell viability was measured with trypan blue staining. Bars represent the mean ± SD of two independent biological experiments. Data have been normalized to untreated cells. Source data are provided as a Source Data file.

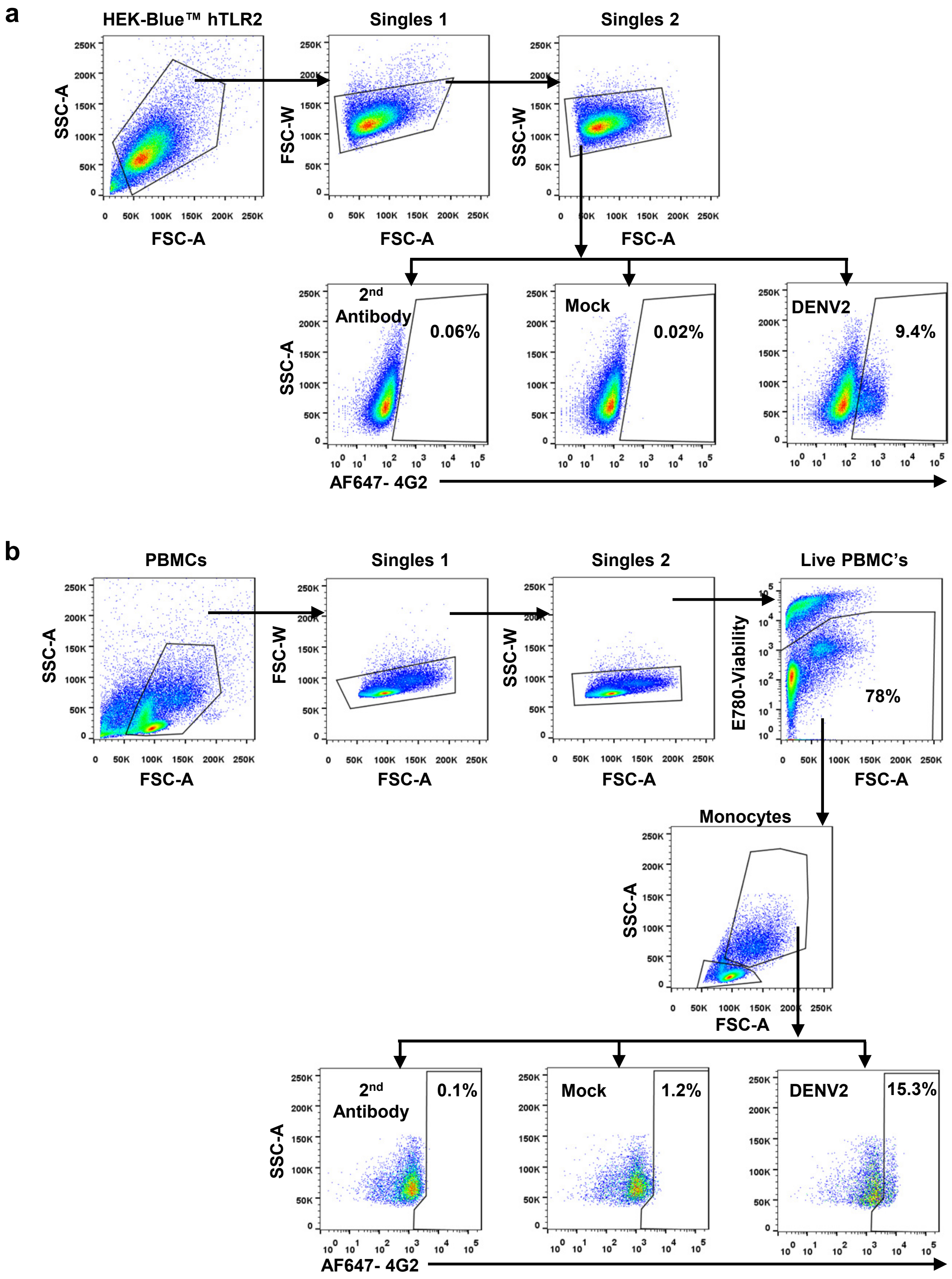


**Supplementary Figure 7**. **Gating strategy used to asses DENV infection.** (**a**) Gating strategy to assess DENV infection in HEK-Blue™ hTLR2 cells as presented in Figure 2e. (**b**) Gating strategy to assess DENV infection in human primary monocytes (within PBMCs) as presented in Figure 2f, Supplementary Fig.8a and 8b.

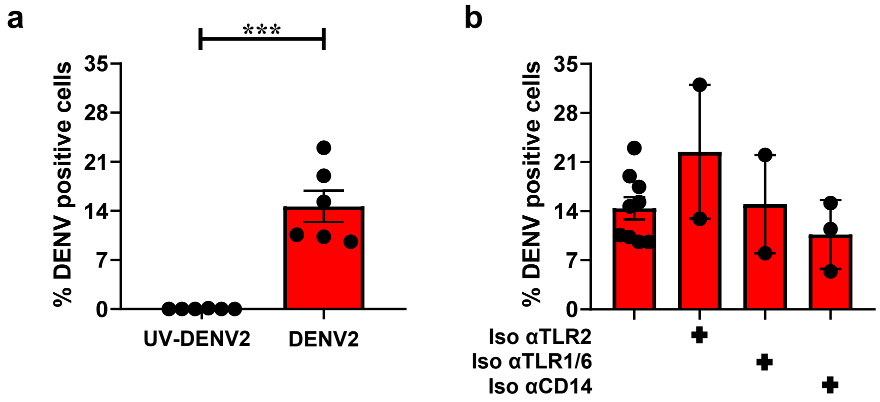


**Supplementary Figure 8. DENV2 but not UV-inactivated DENV2 infects human primary monocytes.** PBMCs from healthy donors were (**a**) exposed to DENV2 at MOI of 20 or its UV- inactivated equivalent (UV-DENV2) for 48h (n=2, two different donors and three different DENV preparations, paired one-tailed t test, ***P<0.001) or (**b**) (mock-) treated with the isotype controls of the αTLR2/1/6 and CD14 prior to infection with DENV2 at the MOI of 20 (n=3, three different donors and up to three different viral preparations). Percentages of DENV-(E) - positive cells were determined by flow cytometry. Data represent the mean ± SEM. Source data are provided as a Source Data file.

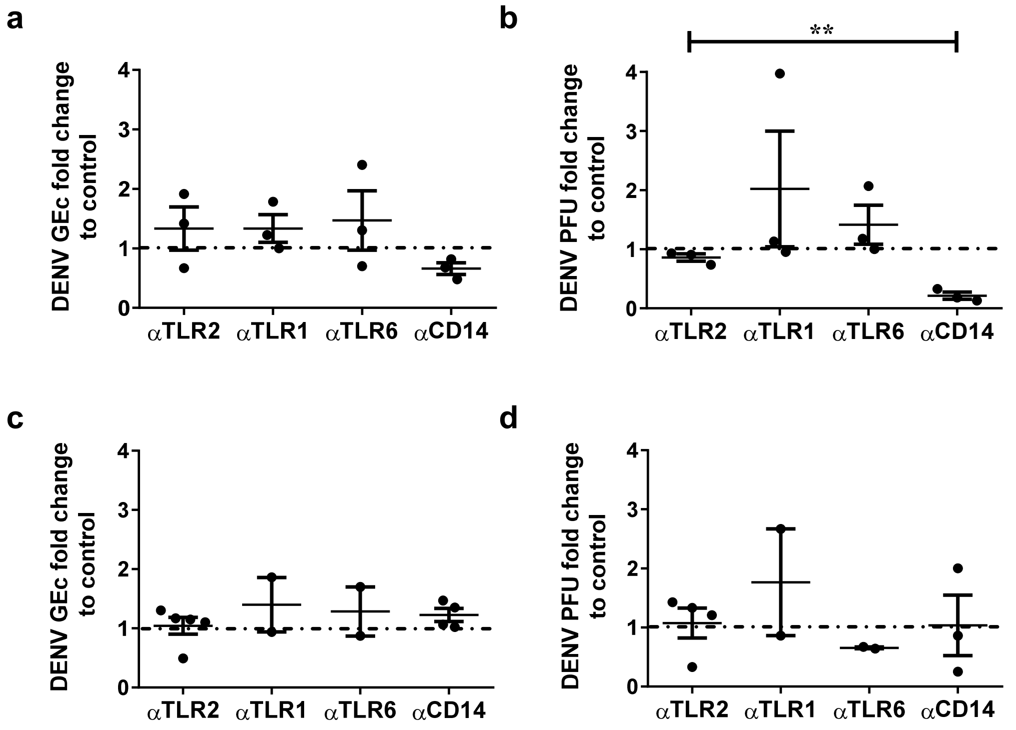


**Supplementary Figure 9. Blockade of TLR2, TLR1, TLR6 or CD14 does not affect virus release.** (**a** and **b**) HEK-Blue™ hTLR2 cells were (mock-)treated with 15 µg/mL of αTLR2, αTLR1, αTLR6 and αCD14 for 2h prior to infection with DENV2 (MOI 10). At 2 hpi, cells were washed, and incubation was continued for 22 hours (n=3, supernatants from three different experiments in HEK-Blue™ hTLR2 cells, paired one-tailed t test, **$P<0.01$). (**c** and **d**) PBMCs from healthy donors were (mock-) treated with αTLR2 or αCD14 (5 µg/mL) for 2h prior to infection with DENV2 at the MOI 10. At 2 hpi, cells were washed, and incubation was continued for 22 hours (**c**) n=5 (supernatants from five different donors), (**d**) n=4 (supernatants from four different donors). (**a** and **c**) Genome equivalent copies (GEc) and (**b** and **d**) Plaque forming units (PFU) were measured as described in Methods section. Bars represent the mean ± SEM. Source data are provided as a Source Data file.

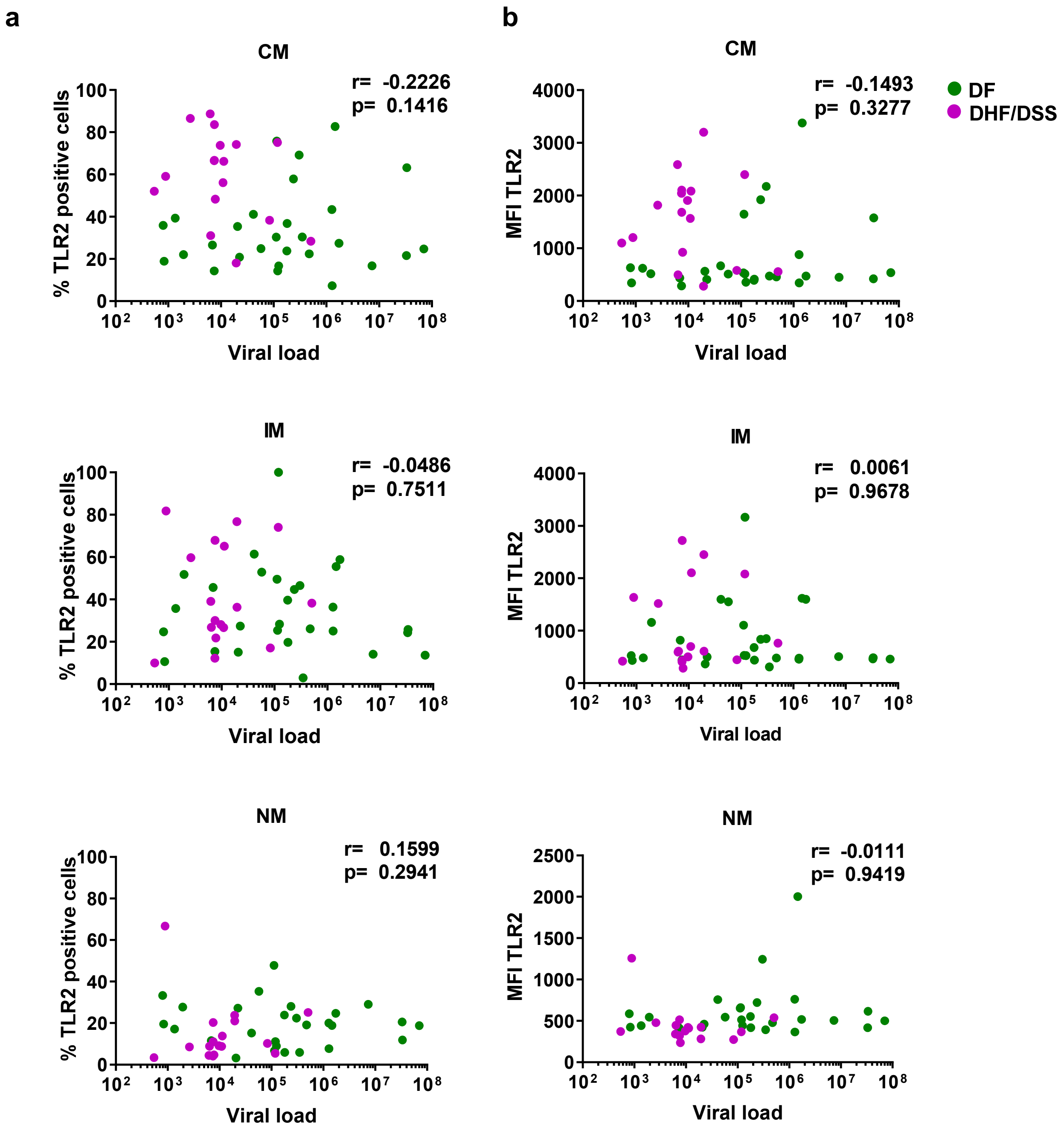


**Supplementary Figure 10. TLR2 expression does not correlate with viral load.** Correlation analysis of (**a**) percentages of TLR2 positive cells and (**b**) mean fluorescence intensity (MFI) of TLR2 expression on monocytes subsets and DENV viral load (determined by qPCR) in our patient's cohort. Green dots represent patients with dengue fever; Magenta dots represent the patients that subsequently developed DHF/DSS. Viral load was measured at hospital admittance within 96h of onset of symptoms. Association was tested by Spearman correlation. Source data are provided as a Source Data file.

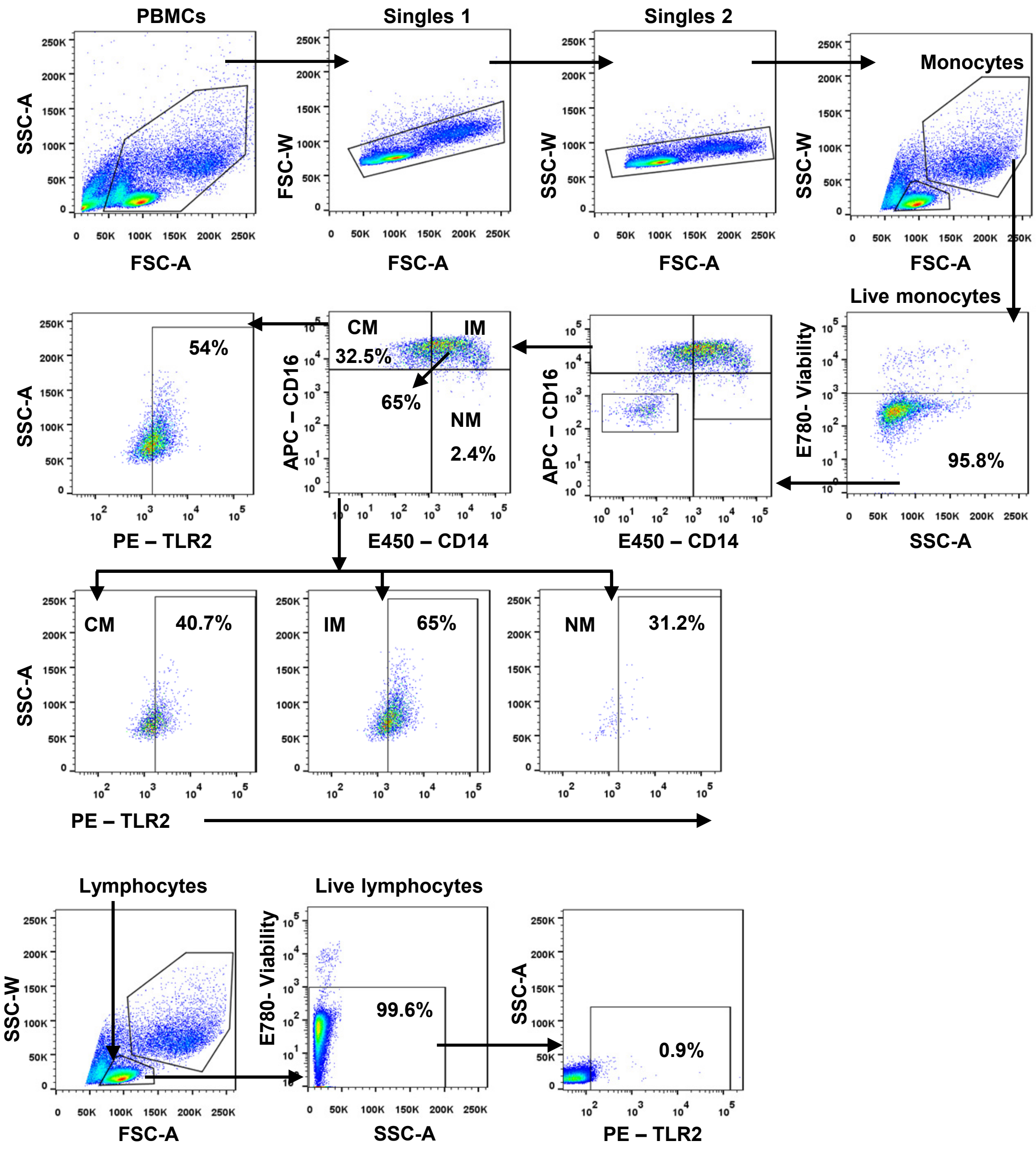


**Supplementary Figure 11**. **Gating strategy used for characterization of monocyte subsets and TLR2 surface expression in monocytes and lymphocytes**. Gating strategy used to characterize monocyte subsets and TLR2 expression in monocytes and lymphocytes from healthy donors as presented in Figure 3a-3d, Supplementary Fig. 12a-12f.

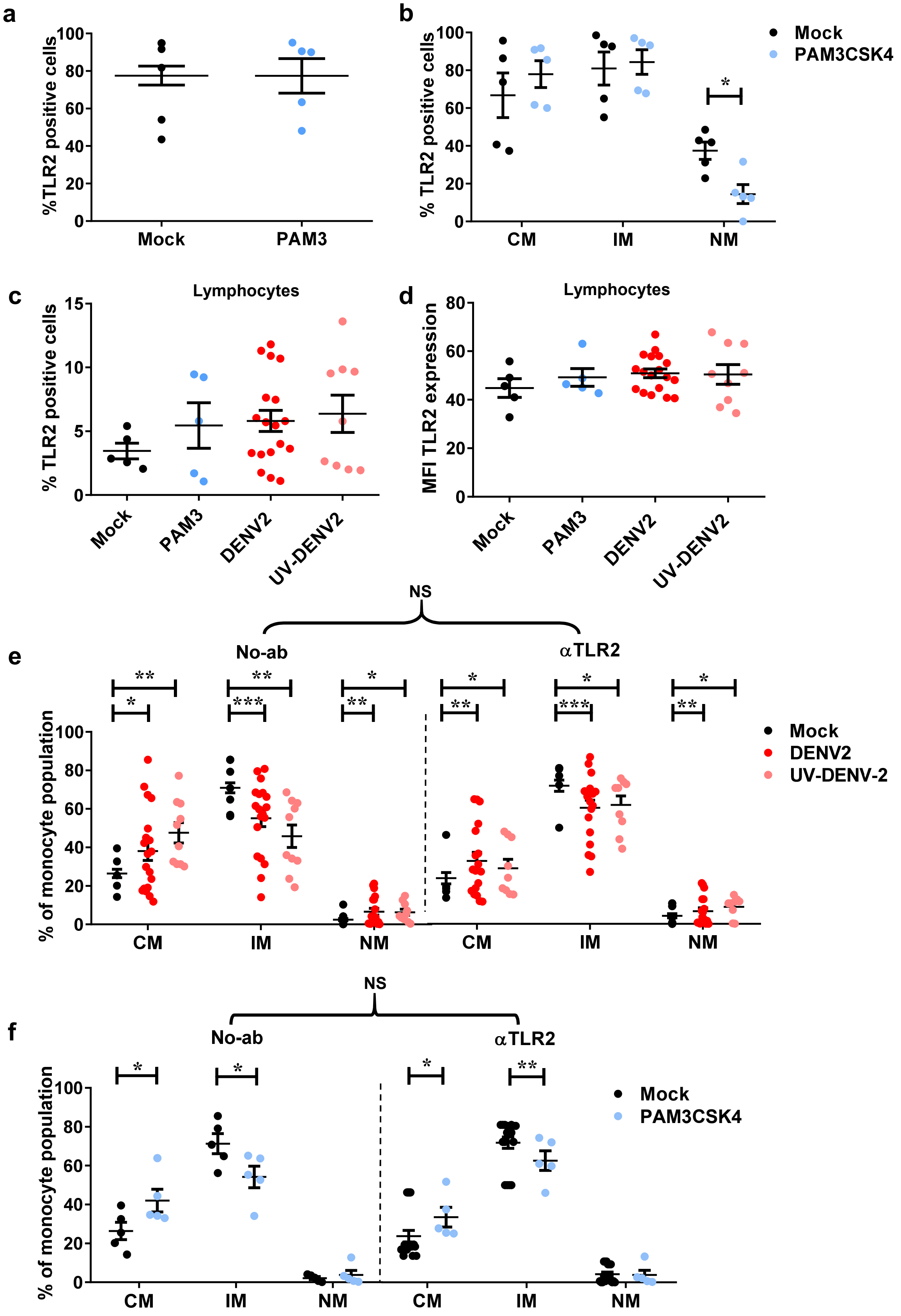


**Supplementary Figure 12. TLR2 expression and frequencies of monocyte subsets after treatment with TLR2 agonists.** PBMCs from healthy donors were (mock-)treated with αTLR2 for 2h prior treatment with PAM3CSK4 (600 ng/mL, DENV2 at MOI of 10 or its UV- inactivated equivalent (UV-DENV2), for 48h. (**a**) TLR2 expression was analyzed for whole monocytes population (n=5) and (**b**) for each monocyte subset after treatment with PAM3CSK4 (n=5, paired one-tailed t test, *P<0.05). (**c**) TLR2 expression was analyzed for the whole lymphocytes in percentages (n=5) and (**d**) MFIs (n=5). (**e**) Frequencies of monocyte subsets as determined in TLR2-blocking and non-blocking conditions after infection with DENV2 and UV-DENV2 (n=5, unpaired one-tailed t test, *P< 0.05, **P<0.01, ***P<0.001) and (**f**) PAM3CSK4 (n=5, paired one-tailed t test, *P< 0.05, **P<0.01). N refers to the number of donors, up to four different viral preparations were used per donor. CM: classical monocytes, IM: intermediate monocytes and NM: non-classical monocytes. Bar represents mean ± SEM. Source data are provided as a Source Data file.

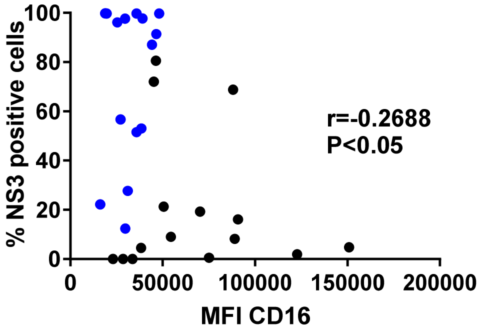


**Supplementary Figure 13. Expression of CD16 expression negatively correlates with infection.** Correlation of mean fluorescence intensity (MFI) of CD16 expression on monocytes (blue dots represent IM, black dots represent NM) and % of DENV infection (NS3) in our patient's cohort. Association was tested by Pearson correlation. Source data are provided as a Source Data file.

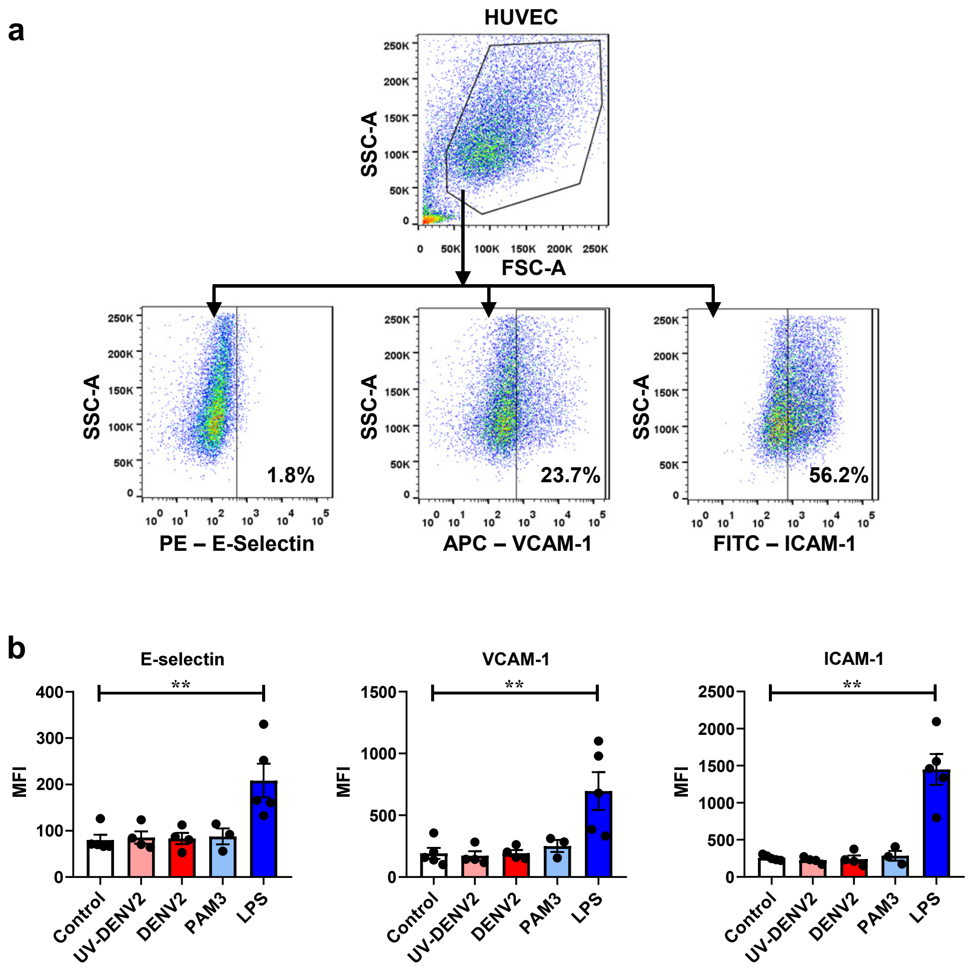


**Supplementary Figure 14. Adhesion molecules expression after 6 hours of incubation with TLR2 and TLR4 agonists. (a)** Gating strategy to determine the expression of adhesion molecules in HUVEC as presented in Figure 4b, Supplementary Fig. 14b, 15a-15d. (**b**) HUVEC were incubated for 6 hours with DENV2 (MOG ~3500), UV-DENV2 (MOG ~3500), PAM3CSK4 (100 ng/mL) and LPS (1 µg/mL) (n=5, paired one-tailed t test, **P<0.01). Surface expression of E-selectin, VCAM-1 and ICAM-1 was determined by flow cytometry. Bar represents mean ±SEM of five independent biological experiments in HUVEC. Source data are provided as a Source Data file.

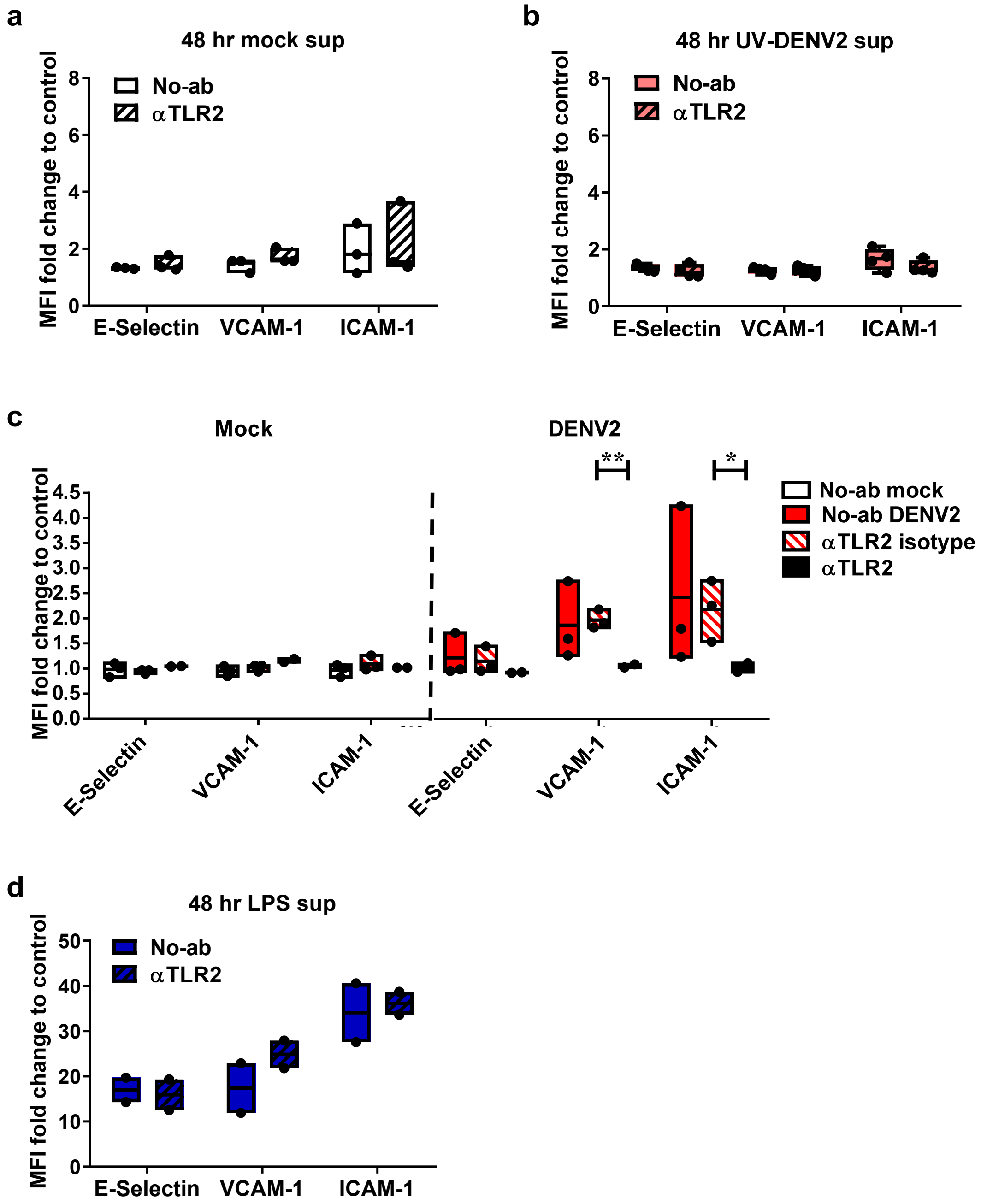


**Supplementary Figure 15. Production of vasoactive mediators by DENV2 infected PBMCs is TLR2 specific.** (**a-d**) HUVEC were incubated for 6 hours with cell-free supernatants from (**a**) mock- (n=3), (**b**) UV-DENV (n=3), (**c**) DENV2 (n=3, unpaired one-tailed t test, *P<0.05, **P<0.001) or (**d**) LPS-treated PBMCs n=2, previously treated with αTLR2 or isotype control blocking antibody. Boxplots show the fold-changes in surface expression of E-selectin, VCAM-1 and ICAM-1 compared to the respective mock of three (**a-c**) or two (**d**) independent biological experiments in HUVEC. The horizontal line represents the median, the top and bottom of the boxes show minimum and maximum. Source data are provided as a Source Data file.

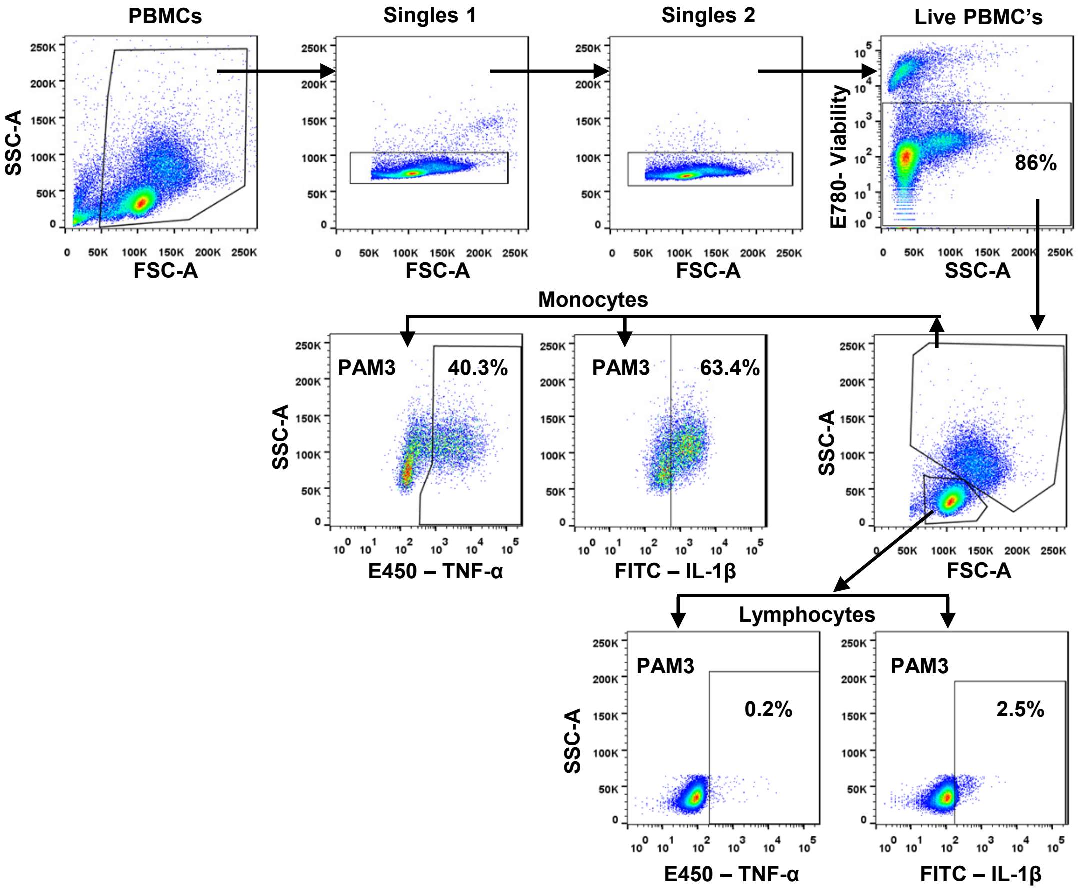


**Supplementary Figure 16**. **Gating strategy to measure the intracellular accumulation of TNF-α and IL-1β in PBMCs.** Gating strategy used to measure the intracellular accumulation of TNF-α and IL-1β in monocytes and lymphocytes within PBMCs as presented in Supplementary Fig. 17a-17f.

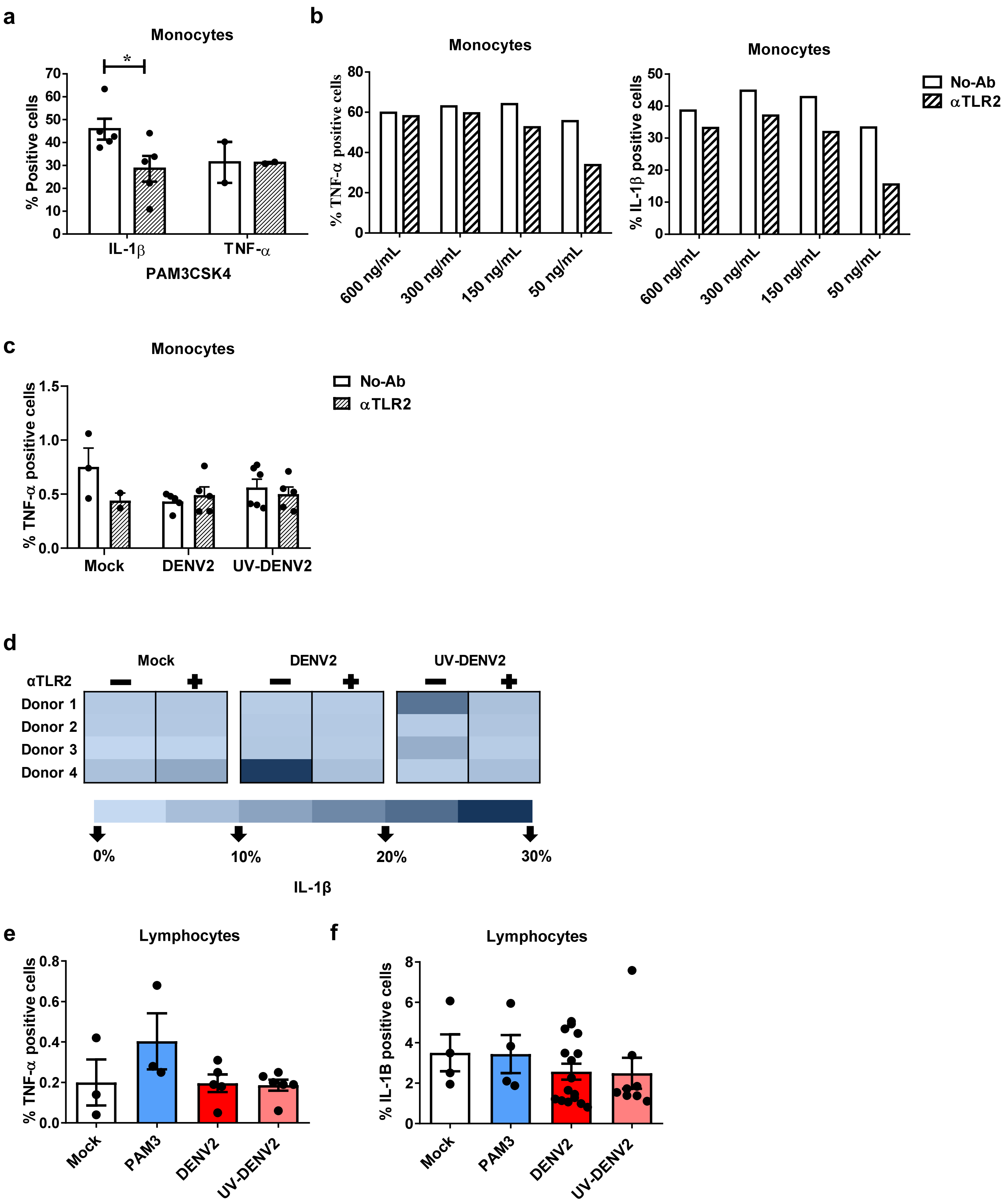


**Supplementary Figure 17**. **Intracellular accumulation of IL-1β and TNF-α in PBMCs after exposure with DENV**. PBMCs from healthy donors were (mock)-treated with αTLR2 (5 µg/mL) for 2h prior treatment with PAM3CSK4 ((**a**) 600 ng/mL, 300 ng/mL, 150 ng/mL, 50 ng/mL), DENV2 (MOI 10) or UV-DENV2 (MOG 1000). (**a-d**) Percentage of monocytes (in PBMCs) with intracellular expression of TNF-α and IL-1β was measured by flow cytometry at 6h and 18h post treatment respectively. (**a**) IL-1β n=5 (five different donors, paired one-tailed t test, *P<0.05), TNF-α n=2 (two different donors). (**b**) TNF-α and IL-1β (one donor). (**c**) TNF-α n=3 (three different donors, two different viral preparation per donor). (**d**) IL-1β n=4 (four different donors. (**e** and **f**) Percentage of lymphocytes with intracellular expression of TNF-α (n=3, three different donors, up to three different viral preparations) and IL-1β (n=4, four different donors, up to three different viral preparations), respectively. Bars represent mean ±SEM. Source data are provided as a Source Data file.

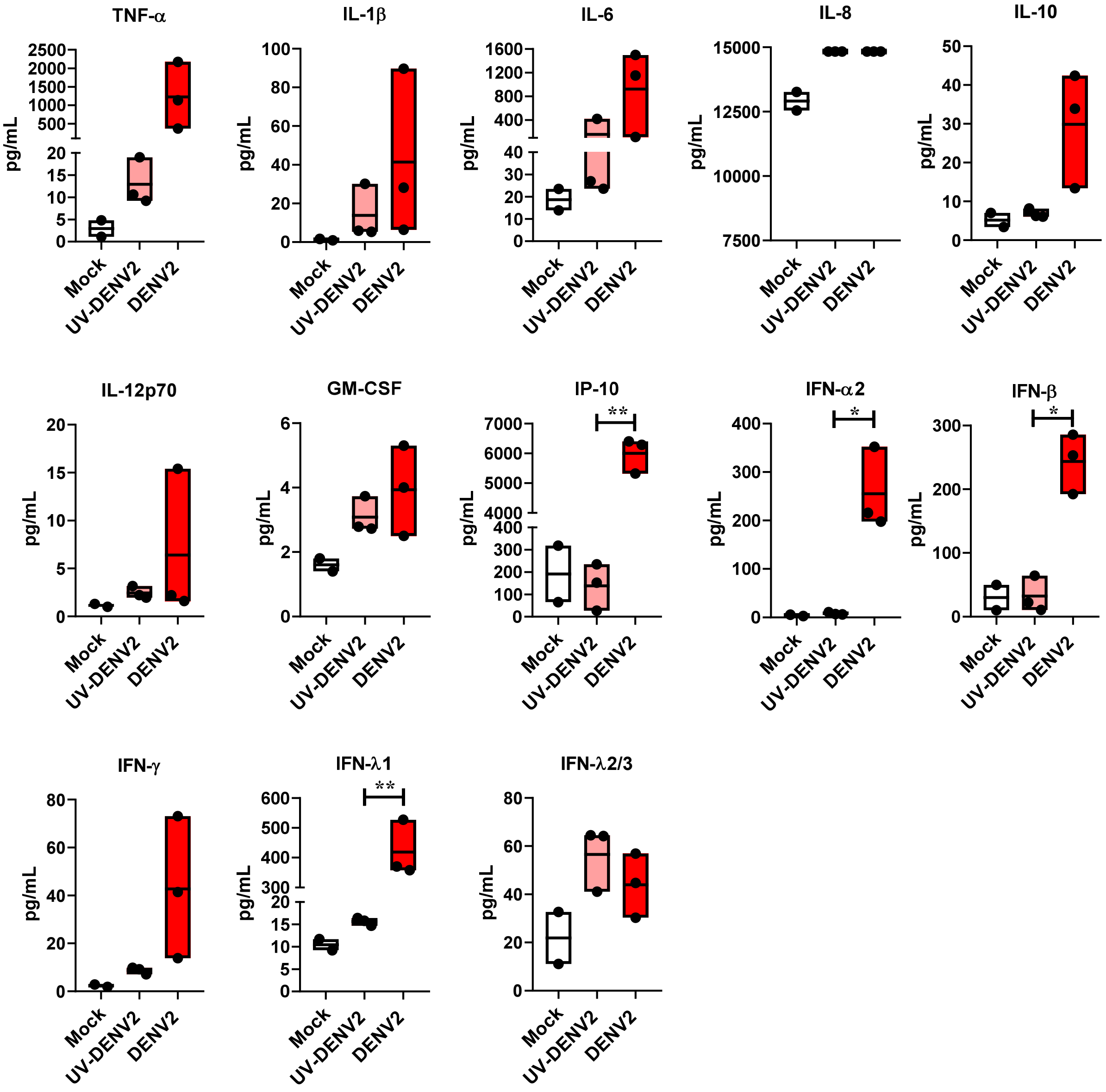


**Supplementary Figure 18. Differential cytokine production by DENV2 and UV-Inactivated DENV2.** PBMCs from healthy donors were (mock) - infected with DENV2 at MOI of 20 or its UV- inactivated equivalent (UV-DENV2) for 48h (n=2, two different donors and three different DENV2/UV-DENV2 preparations, paired one-tailed t test, *$P<0.05$, **$P<0.01$). Cytokine production was measured by flow cytometry using LegendPlex. Each boxplot in the graphs shows the production in picograms per milliliter (pg/mL) of the respective cytokine. The horizontal line represents the mean and the bottom and top of the box show the minimum and maximum values. Source data are provided as a Source Data file.

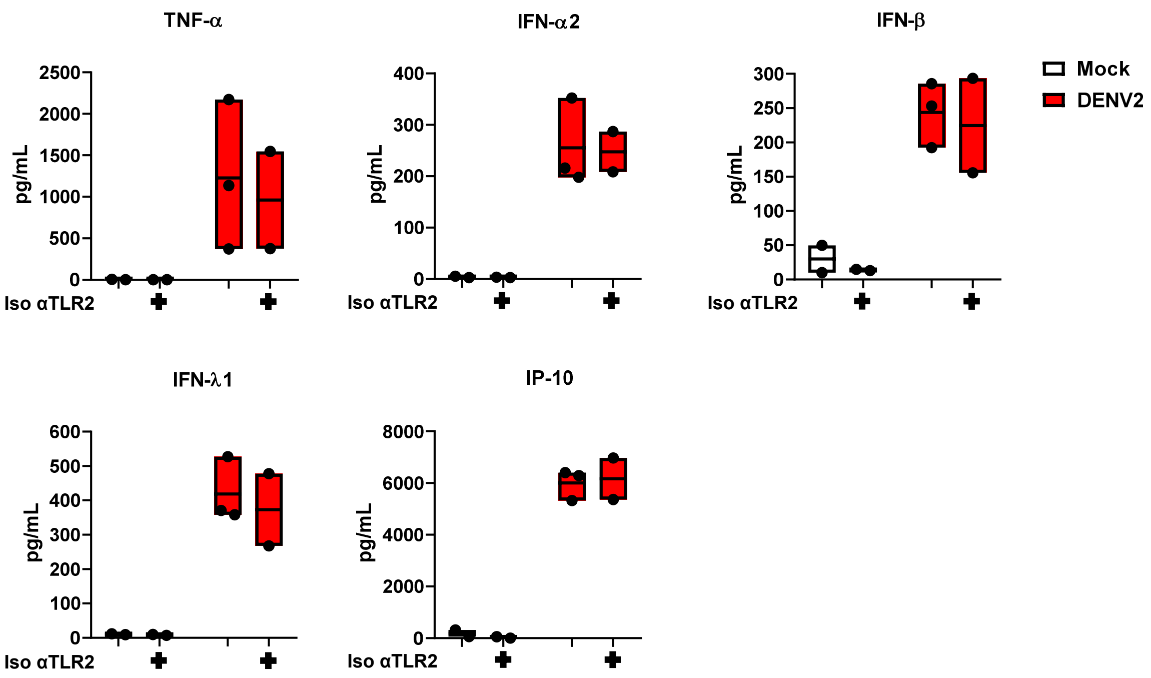


**Supplementary Figure 19. αTLR2 isotype do not impair the DENV2 infection-induced production of cytokines.** PBMCs from healthy donors were (mock) - treated with αTLR2 isotype control (5 µg/mL) for 2h prior to infection with DENV2 at MOI of 20 for 48h (n=2, two different donors and three different DENV2 preparations). Cytokine production was measured by flow cytometry using LegendPlex. Each boxplot in the graphs shows the production in picograms per milliliter (pg/mL) of the respective cytokine. The horizontal line represents the mean and the bottom and top of the box show the minimum and maximum values. Source data are provided as a Source Data file.

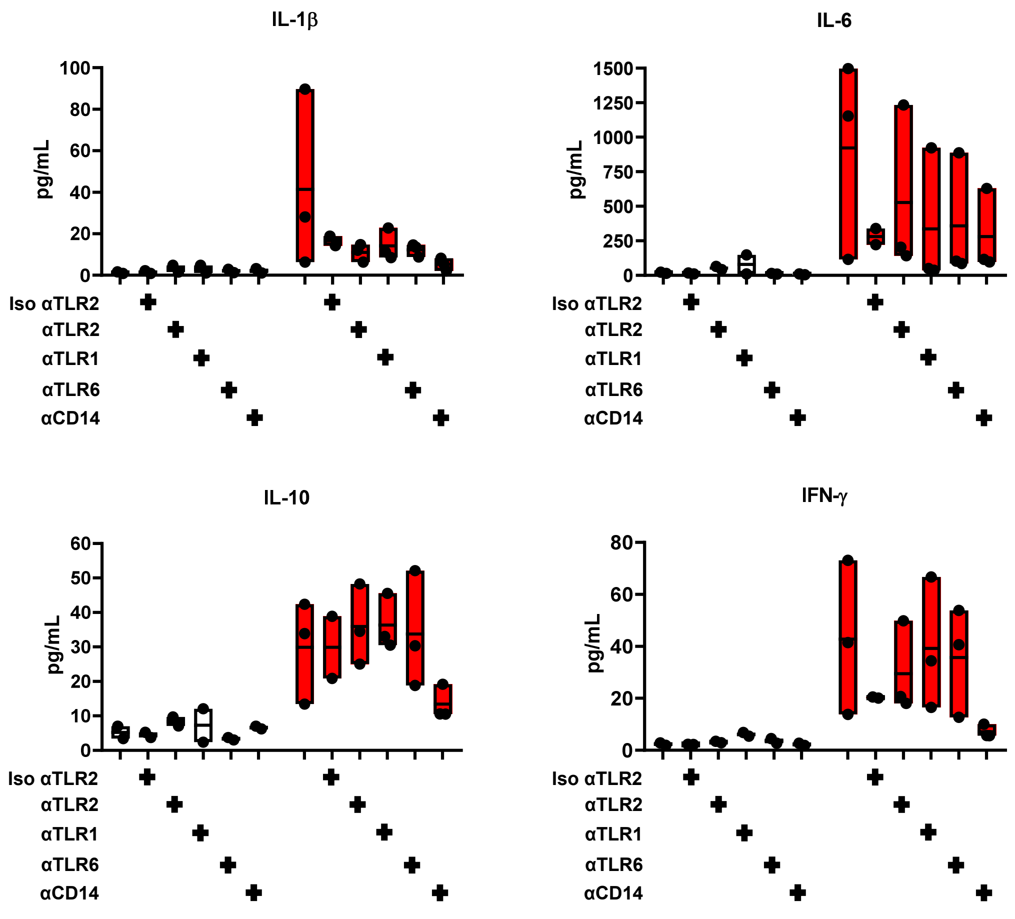


**Supplementary Figure 20. Active DENV2 infection-induced cytokines not in control of TLR2/CD14.** PBMCs from healthy donors were (mock) - treated with αTLR2, αTLR1, αTLR6, αTLR2 isotype control (5 µg/mL) and αCD14 (3 µg/mL) for 2h prior to infection with DENV2 at MOI of 20 (n=2, two different donors and three different DENV2 preparations) for 48h. Cytokine production was measured by flow cytometry using LegendPlex. Each boxplot in the graphs shows the production in picograms per milliliter (pg/mL) of the respective cytokine. The horizontal line represents the mean and the bottom and top of the box show the minimum and maximum values. Source data are provided as a Source Data file.
