# Supplementary material for: TLR2 on blood monocytes senses dengue virus infection and its expression correlates with disease pathogenesis
Source: Nat Commun. 2020 Jun 23;11:3177. doi: 10.1038/s41467-020-16849-7 (PMC7311456; doi:10.1038/s41467-020-16849-7)
Supplement: Supplementary file 3 — Reporting Summary [file 41467_2020_16849_MOESM3_ESM.pdf]

nature research

Corresponding author(s): Izabela A. Rodenhuis-Zybert
Tineke Cantaert

Reporting Summary

Nature Research wishes to improve the reproducibility of the work that we publish. This form provides structure for consistency and transparency in reporting. For further information on Nature Research policies, see Authors & Referees and the Editorial Policy Checklist.

Statistical parameters

When statistical analyses are reported, confirm that the following items are present in the relevant location (e.g. figure legend, table legend, main text, or Methods section).

n/a	Confirmed	
☐	☒	The exact sample size (*n*) for each experimental group/condition, given as a discrete number and unit of measurement
☐	☒	An indication of whether measurements were taken from distinct samples or whether the same sample was measured repeatedly
☐	☒	The statistical test(s) used AND whether they are one- or two-sided<br>*Only common tests should be described solely by name; describe more complex techniques in the Methods section.*
☒	☐	A description of all covariates tested
☐	☒	A description of any assumptions or corrections, such as tests of normality and adjustment for multiple comparisons
☐	☒	A full description of the statistics including central tendency (e.g. means) or other basic estimates (e.g. regression coefficient) AND variation (e.g. standard deviation) or associated estimates of uncertainty (e.g. confidence intervals)
☐	☒	For null hypothesis testing, the test statistic (e.g. *F*, *t*, *r*) with confidence intervals, effect sizes, degrees of freedom and *P* value noted<br>*Give P values as exact values whenever suitable.*
☒	☐	For Bayesian analysis, information on the choice of priors and Markov chain Monte Carlo settings
☒	☐	For hierarchical and complex designs, identification of the appropriate level for tests and full reporting of outcomes
☐	☒	Estimates of effect sizes (e.g. Cohen's *d*, Pearson's *r*), indicating how they were calculated
☐	☒	Clearly defined error bars<br>*State explicitly what error bars represent (e.g. SD, SE, CI)*

Our web collection on statistics for biologists may be useful.

Software and code

Policy information about availability of computer code

Data collection	BD FACSCanto II: Data acquisition was done using BD FACSDiva software (BD Bioesceinces).<br>BD FACSVerse: Data acquisition was done using BD FACSuite software v1.0.6.5230 (BD Bioesceinces).<br>MACSQuant: Data acquisition was done using MACSQuantify Software (Miltenyi Biotec).
Data analysis	Data and statistical analysis was performed using Prism 6.01 (Graphpad, USA)<br>Cytokine/chemokine analysis was performed using LEGENDplex v8.0 (BioLegend).<br>Flowjo software X 10.0.7r2(BD Biosciences).

For manuscripts utilizing custom algorithms or software that are central to the research but not yet described in published literature, software must be made available to editors/reviewers upon request. We strongly encourage code deposition in a community repository (e.g. GitHub). See the Nature Research guidelines for submitting code & software for further information.

Data

Policy information about availability of data

All manuscripts must include a data availability statement. This statement should provide the following information, where applicable:

- Accession codes, unique identifiers, or web links for publicly available datasets
- A list of figures that have associated raw data
- A description of any restrictions on data availability

All data generated and analyzed in this study are included in the manuscript (and its supplementary files). The source data underlying Figs. 1A-E, 2A–G, 3A-D, 4B-D, 5 and Supplementary Figs. 3, 4A-B, 5A-D, 6, 8A-B, 9A-D, 10A-B, 12A-F, 13, 14B, 15A-D, 17A-F, 18, 19 and 20 are provided as a Source Data file.

Field-specific reporting

Please select the best fit for your research. If you are not sure, read the appropriate sections before making your selection.

- [x] Life sciences
- [ ] Behavioural & social sciences
- [ ] Ecological, evolutionary & environmental sciences

For a reference copy of the document with all sections, see nature.com/authors/policies/ReportingSummary-flat.pdf

Life sciences study design

All studies must disclose on these points even when the disclosure is negative.

Sample size	Sample size was determined based on the availability of samples from dengue patients classified for severity of dengue disease as per criteria specified by the World Health Organization (1997). Samples were collected during the study from June 2016 to July 2017. 15 age-matched healthy controls were included in the study.<br>For in-vitro experiments we reasoned that a sample size of at least 3 independent biological experiments (n=3) would be sufficient as is a standard practice
Data exclusions	Exclusion criteria were pre-established. Patient samples with insufficient cell counts as determined by flow cytometry were excluded from the study.
Replication	Reproducibility of the data was improved by increasing the sample size. Experiments on human participants (dengue positive patients) could not be replicated due to limited availability of patient biological material.<br>All in-vitro experiments were standardized and performed at least by two scientist three times to assure reproducibility, all attempts at replication were successful. In-vitro experiments using human PBMCs are prone to donor variation, to improve reproducibility we increased sample size. PBMCs from different donors were treated independently and all attempts at replication were successful.
Randomization	Samples were allocated randomly into experimental groups
Blinding	Investigators were blinded to group allocations during data collection and analysis of sample data.

Reporting for specific materials, systems and methods

Materials & experimental systems

n/a	Involved in the study
[ ]	[x] Unique biological materials
[ ]	[x] Antibodies
[ ]	[x] Eukaryotic cell lines
[x]	[ ] Palaeontology
[x]	[ ] Animals and other organisms
[ ]	[x] Human research participants

Methods

n/a	Involved in the study
[x]	[ ] ChIP-seq
[ ]	[x] Flow cytometry
[x]	[ ] MRI-based neuroimaging

Unique biological materials

Policy information about availability of materials

Obtaining unique materials	All patients presenting with dengue-like symptoms were recruited in the study followed by confirmation of dengue infection by diagnostic tests. Samples from patients positive for dengue and healthy age-matched controls were included in the study. Ethical

approval was obtained from the National Ethics Committee of Health Research of Cambodia. Written informed consent was obtained from all participants or the guardians of participants under 18 years of age before inclusion in the study. As specified in the material & methods section of the manuscript

Antibodies

Antibodies used

anti-human CD19-AF488(1:50, BioLegend, #302219; clone HIB19; lot B190831)
anti-human TLR2-PE (1:25, BioLegend, #309707; clone TL2.1; lot B195155)
isotype matched control labelled with PE (1:25, BioLegend, #400211; clone MOPC-173; lot B227641)
anti-human CD4-PerCP-Cy5.5 (1:50, BioLegend, #317428; clone OKT4; lot B247735)
anti-human CD14-APC (1:50,BioLegend, #467117; clone 63D3; lot B230116)
anti-human CD16 PE-Cy7 (1:250, BioLegend, #302016; clone 3G8; lot B262406)
anti-human CD45RO APC-Cy7 (1:25, BioLegend, #304228; clone UCHL1; lot B236646)
anti-human CD56 BV421 (1:25, BioLegend, #362552; clone 5.1H11; lot B249401)
anti-human CD3 BV510 (1:50, BioLegend, #317332; clone OKT3; lot B226707)
rabbit polyclonal anti-dengue NS3 antibody (10µg/mL, GeneTex; GTX124252; lot 42508)
goat anti-rabbit antibody with AlexaFluor 488 (1:500, Abcam; #ab150077; lot GR239034-3)
rabbit polyclonal isotype-matched control (10µg/mL, BioLegend; #910801; clone Poly29108; lot B214550)

Fixable Viability Dye eFluor 780 (1:500, eBioscience, #65-0865-14; lot 1923272)
CD282 (TLR2) Monoclonal Antibody PE (1:10, eBioscience, clone TL2.1, #12-9922-41)
CD14 Monoclonal Antibody (61D3) eFluor 450 (1:20, eBioscience, clone 613D, #48-0149-41; lot 4312909)
CD16 Monoclonal Antibody APC (1:20, eBioscience, clone CB16, #17-0168-41; lot 4294062)
TNF alpha Monoclonal Antibody eFluor 450 (1:10, eBioscience, clone MAb11; #48-7349-41)
IL-1 beta Monoclonal Antibody FITC (1:5, eBioscience, clone B-A15; #BMS127FI; lot 191796000)
Mouse IgG2a kappa Isotype Control PE (1:10, eBioscience, clone eBM2a, #12-4724-81)
Mouse IgG1 kappa Isotype Control eFlour 450 (1:20, eBioscience, clone P3.6.2.8.1; #48-4714-82; lot 4287228)
Mouse IgG1 kappa Isotype Control APC (1:20, eBioscience, clone P3.6.2.8.1; #17-4714-81; lot 4276174)
Mouse IgG1 kappa Isotype Control FITC (1:5, eBioscience, clone P3.6.2.8.1; #11-4714-81)
PE anti-human CD62E Antibody (1:100, Biolegend, clone HCD62E; #322606)
APC anti-human CD106 Antibody (1:100, Biolegend, clone STA; #305810)
FITC anti-human CD54 Antibody (1:100, Biolegend, clone HCD54; #322720)
PE anti-mouse IgG2a Antibody (1:100, Biolegend, clone RMG2a-62; #407107)
APC anti-mouse IgG1 Antibody (1:100, Biolegend, clone RMG1-1; #406609)
FITC anti-mouse IgG1 Antibody (1:100, Biolegend, clone RMG1-1; #406605)
anti-Flavivirus Group Antigen Antibody (1:800, Millipore, clone D1-4G2-4-15; #MAB10216)
rabbit anti-mouse IgG-coupled to AF647 (1:200, Thermofisher, # A-21446)
Anti-hTLR2-IgA (5 µg/mL (PBMC's) and 15 µg/mL (HEK's),InvivoGen, clone B4H2; #maba2-htlr2)
PAb-hTLR6 (5 µg/mL (PBMC's and 15 µg/mL (HEK's), InvivoGen, #pab-hstlr6)
PAb-hTLR1 (5 µg/mL (PBMC's) and 15 µg/mL (HEK's), InvivoGen, #pab-hstlr1)
anti-hCD14-IgA (5 µg/mL (PBMC's) and 15 µg/mL (HEK's), InvivoGen, clone D3B8; #maba-hcd14)
Human IgA2 Isotype Control (5 µg/mL (PBMC's) and 15 µg/mL (HEK's), anti-βGal, InvivoGen, clone T9C6, #maba2-ctrl)
normal rat PAb IgG control (5 µg/mL (PBMC's) and 15 µg/mL (HEK's), InvivoGen, #pab-sctr)
anti-hCD14-IgG (5 µg/mL (PBMC's), InvivoGen, clone D3B8; #mabg-hcd14),
Human IgG1 Isotype control (5 µg/mL (PBMC's), InvivoGen, Anti-β-Gal; #bgal-mab1)
LEGENDplex Human anti-virus response panel with V-bottom (13-plex, BioLegend, #740390, Lot#B262965)

Validation

All antibodies used in the study were validated by the manufacturer and validation can be found on manufacturer's website supported by multiple publications.
The rabbit polyclonal anti-dengue NS3 antibody and the mouse anti-DENV E antibody were validated in house by flow cytometry testing with isotype control or secondary antibody stainings, absence of staining after infection of target cells with non-infectious dengue virus particles and MOI-dependent increase in percentage of virus-infected target cells.

Eukaryotic cell lines

Policy information about cell lines

Cell line source(s)

HEK-Blue hTLR2, HEK-Blue hTLR4 and HEK-Blue Null2 were obtained from InvivoGen.
Vero E6 (ATCC: CRL-1586, a kind gift from Gorben Pijlman (Wageningen University, Wageningen, the netherlands) and Vero-WHO cells (European Collection of Cell Culture # 88020401) were a kind gift from Michael S. Diamond. (Washington University, Sr. Louis, USA)
Aedes albopictus C6/36 cells (ATCC: CRL-1660) and Baby hamster kidney-21 cells clone 15 (BHK-15, not commercially available) were a kind gift from Richard Kuhn (ATCC: CRL-1660, Purdue University, West Lafayette, Indiana, USA)
HUVEC were obtained from Lonza (the Netherlands).

Authentication	The cell lines were not authenticated.<br>HEK-Blue reporter cells were cultured and tested using manufacturer's instructions, every cell responded specifically to its respective TLR agonist.
Mycoplasma contamination	All of the cell lines and viral preparations used in this study were tested negative for Mycoplasma contamination using a commercial functional method (Lonza, the Netherlands) and/or in-house qPCR assay adapted from Baronti et al, 2013 (Mycoplasma removal: Simple curative methods for viral supernatants)
Commonly misidentified lines<br>(See ICLAC register)	No commonly misidentified cell lines were used in the study.

Human research participants

Policy information about studies involving human research participants

Population characteristics	See Table 1 of the manuscript
Recruitment	Suspected DENV patients were consecutively enrolled and a blood sample was drawn for DENV diagnosis at hospital admittance. In total, 110 subjects were enrolled. Patients were diagnosed as acute DENV-infected as following: a positive qRT-PCR or NS1 positive by rapid test at hospital admission, or seroconversion from DENV-IgM negative to IgM positive during the hospital stay (admittance and discharge sample). Patients who were DENV-IgM positive and RTPCR/NS1 negative at admission were excluded from analysis. Of the 110 patients enrolled, 21 were negative for DENV diagnostics, 12 were positive for DENV IgM, and negative for qRT-PCR and/or NS1 at admission and hence excluded (see above), and 3 subjects were excluded due to lack of follow up sample at discharge. Of 20 subjects, samples were not available for analysis. This resulted in a cohort of 54 confirmed acute DENV-infected cases. Patients were followed up during their hospital stay and WHO 1997 classification was done after hospital discharge. 32 subjects were classified as DF, 22 as DHF and 10 as DSS patients. Ethical approval was obtained from the National Ethics Committee of Health Research of Cambodia. Written informed consent was obtained from all participants or the guardians of participants under 18 years of age before inclusion in the study. There was no self-selection bias.

Flow Cytometry

Plots

Confirm that:

- [x] The axis labels state the marker and fluorochrome used (e.g. CD4-FITC).
- [x] The axis scales are clearly visible. Include numbers along axes only for bottom left plot of group (a 'group' is an analysis of identical markers).
- [x] All plots are contour plots with outliers or pseudocolor plots.
- [x] A numerical value for number of cells or percentage (with statistics) is provided.

Methodology

Sample preparation	Patients: Peripheral blood mononuclear cells (PBMCs) were isolated from dengue patients by Ficoll Histopaque separation as described in Materials and Methods section. Briefly, PBMCs were washed with PBS/BSA and surface staining was performed with the antibody mix. For intracellular staining, PBMCs were fixed and permeabilized using True nuclear transcription factor buffer set (BioLegend) and stained intracellularly using a rabbit anti-DENV NS3 antibody (GeneTex) followed by goat anti-rabbit IgG conjugated with AF488 (Abcam).<br><br>In-vitro assays:<br>PBMCs from healthy donors were isolated by Ficoll Histopaque separation. PBMCs were washed with PBS/EDTA/FBS and surface staining was performed with the antibody mix as described in Materials and Methods section. For intracellular staining, PBMCs were fixed with 4% paraformaldehyde and permeabilized using and PBS/Tween 20. Intracellular staining was performed using TNF-α e450 and IL-1β FITC primary antibodies (eBioscience). For cytokine and chemokine determination, cell-free supernants were analyzed by LEGENDplex Human anti-virus response panel with V-bottom (BioLegend).<br><br>HEK-Blue hTLR2 cells were washed with PBS/EDTA/FBS and surface staining was performed with the antibody mix as described in Materials and Methods section. HEK-Blue hTLR2 cells were fixed with 4% paraformaldehyde and permeabilized using and PBS/Tween 20. Intracellular staining was performed using a mouse anti-DENV E (Millipore) followed by rabbit anti-mouse IgG AF647 (Molecular probes).<br><br>HUVEC cells were washed with PBS/EDTA/FBS and surface staining was performed with the antibody mix as described in Materials and Methods section. Briefly, cells were stained for adhesion molecules E-selectin, VCAM-1 and ICAM-1 (BioLegend) and then fixed with 4% paraformaldehyde.
Instrument	BD FACSCanto II (BD Biosciences) (Cat. No. 338962, Laser configuration 4-2-2) was used to analyze PBMCs from dengue patients<br>BD FACSVerse flow cytometer (BD Biosciences, Cat. No. 651155, Laser configuration 4-2-2) or MACSQuant (Miltenyi Biotec, SN: 2215, Laser configuration 4-2-1) were used to analyze for in-vitro experiments.
Software	BD FACSCanto II: Data acquisition was done using BD FACSDiva software (BD Bioseinces).<br>BD FACSVerse: Data acquisition was done using BD FACSuite software v1.0.6.5230 (BD Bioseinces).

MACSQuant: MACSQuantify Software (Miltenyi Biotec).
Cytokine and chemokine data were analyzed using LEGENDplex v8.0 software (BioLegend).
Analysis of flow cytometry data was done using FlowJo X 10.0.7r2 software.

Cell population abundance

Patient PBMCs: 30, 000 single cells were analyzed. Total PBMC counts for patient samples were variable. Monocytes (CD56 negative, CD14 and CD16 positive cells,) comprised around 10-15 % of this population.
For In-vitro experiments using PBMCs: 40, 000 live cells were analyzed. Monocytes (CD14 and CD16 positive cells) comprised around 10-15% of this population.
For In-vitro experiments using HUVEC: 20, 000 cells were analyzed.
For In-vitro experiments using HEK-Blue hTLR2 cells: 60,000 cells were analyzed.

Gating strategy

Patients: PBMC populations from patient samples were defined by plotting SSC-A vs FSC-A. To ensure that only single cells were analyzed, FSC-A vs FSC-H plot was used to exclude doublets or cell aggregates. Thereafter, to exclude B cells and T cells, single cells were plotted CD3 (AmCyan) vs CD19 (FITC) and CD3-CD19- cells were gated. To exclude NK cells, CD3-CD19- cells were gated SSC-A vs CD56 (Pacific Blue). Cells were then plotted CD14 (APC) vs CD16 (PE-Cy7) to define monocyte subsets. TLR2 expression was determined for classical monocytes and intermediate monocytes (CD14 vs TLR2) and non-classical monocytes (CD16 vs TLR2). Isotype control was used to define positive TLR2 cells (SSC-A vs Isotype PE). TLR2 expression was also determined in the live lymphocyte fraction (SSC-A vs TLR2).

For detection of DENV infection in patient monocytes, PBMCs from patient samples were divided into two parts after fixation and permeabilization - one part for staining with rabbit anti-DENV NS3 primary and goat anti-rabbit secondary antibody and other part for staining with goat anti-rabbit secondary antibody only. PBMC populations from patient samples were defined by plotting SSC-A vs FSC-A and gated for monocytes. To ensure that only single cells were analyzed, FSC-A vs FSC-H plot was used to exclude doublets or cell aggregates. Thereafter, cells from the monocyte fraction were plotted CD14 (APC) vs CD16 (PE-Cy7) to define monocyte subsets. DENV infection was detected using anti-DENV NS3 primary antibody with secondary antibody conjugated to AF488. Samples stained with AF488-conjugated goat anti-rabbit secondary antibody was used to define NS3+ dengue-infected cells. Rabbit isotype control was used as a staining control.

For In-vitro experiments using PBMCs: PBMCs populations were defined by plotting SSC-A vs FSC-A. To ensure that only live single cells were analyzed, FSC-W vs FSC-A and SSC-W vs FSC-a plots were used to exclude doublets or cell aggregates. Thereafter, SSC-A vs FSC-A plot was used again to gate all PBMCs populations. Dead cells (total PBMCs, monocyte fraction or lymphocyte fraction) were further excluded by gating on the cells negative for the fixable Viability Dye eFluor 780 (eFluor 780 vs SSC-A). Live cells from the monocyte fraction were plotted CD14 (eF450) vs CD16 (APC) to define monocyte subsets. Isotype controls were used to define positive CD14 (Iso eF450 vs SSC-A) and CD16 (SSC-A vs Iso APC) cells. TLR2 expression was determined for monocytes in general or in each monocyte subset (SSC-A vs TLR2). Isotype control was used to define positive TLR2 cells (SSC-A vs Isotype PE). TLR2 expression was also determined in the live lymphocyte fraction (SSC-A vs TLR2). For the intracellular detection of IL-1β and TNF-α, the live monocyte fraction was plotted SSC-A vs TNF-α (eF450) and/or IL-1β (FITC). Isotype controls were used to define positive IL-1β (SSC-A vs Iso FITC) and TNF-α (SSC-A vs Iso eF450) expressing cells. For detection of DENV infection, dengue virus positive live monocytes were detected by plotting SSC-A vs mouse anti-DENV E ab 4G2 (AF 647). Non-infected cells were gated by plotting SSC-A vs rabbit anti-mouse IgG AF647 secondary antibody. UV-inactivated DENV was used to validate the results.

For in-vitro experiments using HEK-Blue-hTLR2 cells: HEK-Blue-hTLR2 cell population were defined by plotting SSC-A vs FSC-A. To ensure that only single cells WERE be analyzed, FSC-W vs FSC-A and SSC-W vs SSC-a plots were used to exclude doublets or cell aggregates. Dengue virus positive cells were detected by plotting SSC-A vs mouse anti-DENV E ab 4G2 (AF 647). Non-infected cells were gated by plotting SSC-A vs rabbit anti-mouse IgG AF647 secondary antibody.

For in-vitro experiments using HUVEC: HUVEC cells were defined by plotting SSC-A vs FSC-A. E-selectin (PE), VCAM-1 (APC) and ICAM-1 (FITC) positive cells were plotted using SSC-A vs E-selectin, SSC-A vs VCAM-1, SSC-A vs ICAM-1. Isotype controls were used to define positive E-selectin (SSC-A vs Iso PE), VCAM-1 (SSC-A vs Iso APC) and ICAM-1 (SSC-A vs Iso FITC) cells.

☒ Tick this box to confirm that a figure exemplifying the gating strategy is provided in the Supplementary Information.
